# Effects of Antimicrobial Peptides on the Growth Performance of Squabs Were Investigated Based on Microbiomics and Non-Targeted Metabolomics

**DOI:** 10.3390/ani15213099

**Published:** 2025-10-25

**Authors:** Lihuan Deng, Yingying Yao, Haiying Li, Qingqing Lu, Run Wu

**Affiliations:** College of Animal Science, Xinjiang Agricultural University, Urumqi 830091, China; dlh2498217132@163.com (L.D.); yyy1234560317@163.com (Y.Y.); 17590813280@163.com (Q.L.); 18555384822@163.com (R.W.)

**Keywords:** antimicrobial peptides, squab, growth performance, intestinal health, metabolites, microbiota

## Abstract

**Simple Summary:**

In this study, the molecular mechanisms of antimicrobial peptides (AMPs) on the growth performance of squabs were investigated by using microbiome and non-targeted metabolomics techniques. The results showed that dietary supplementation of 200 mg/kg AMPs could improve the growth performance, liver antioxidant capacity and intestinal morphology of pigeons. The increase in the abundance of *Lactobacillus* and the regulation of key metabolic pathways and metabolites provide rich information for a deeper understanding of the mechanism by which AMPs affect the growth performance of squabs. However, the specific molecular mechanisms and physiological regulatory networks behind these changes remain to be elucidated by further studies.

**Abstract:**

This study aims to investigate the effects of dietary supplementation with AMPs on the growth performance, antioxidant capacity, and intestinal health of squabs. Furthermore, metagenomic and metabolomic approaches were employed to identify key differential bacterial species and metabolites associated with growth performance, and thereby the potential mechanisms underlying the enhancement of squab growth and development by AMPs being elucidated. One hundred and twenty pairs of healthy adult White Carneau pigeons (2 years old) were randomly divided into two groups, the control group (CK, fed with basal diet) and antimicrobial peptide group (AP, fed with basal diet +200 mg/kg antimicrobial peptide), with 10 replicates per group and 6 pairs of breeding pigeons per replicate. The experiment lasted for 53 days, including 7 days of prefeeding, 18 days of incubation and 28 days of feeding. In this study, squabs were weighed at 0 and 28 days of age to evaluate growth performance. At 28 days of age, duodenal contents were collected to assess digestive enzyme activities, while jejunal and liver tissues were harvested to determine antioxidant capacity. Intestinal morphology was examined using tissue samples from the duodenum, jejunum, and ileum. Finally, ileal contents were collected for a comprehensive analysis of microbial composition and metabolite profiles in the two experimental groups, employing high-throughput sequencing and LC-MS/MS techniques. The results showed that body weight, liver total antioxidant capacity (T-AOC), jejunal malondialdehyde (MDA) content, jejunum and ileum villus height-to-crypt depth ratio (VH/CD) were significantly increased, and jejunal crypt depth (CD) was significantly decreased in the AP group at 28 days of age (*p* < 0.05). In addition, the microbiome data showed that *Lactobacillus* in the AP group was a biomarker with significant differences (*p* < 0.05). Metabolomics analysis showed that the steroid hormone biosynthesis pathway was significantly different between the two groups (*p* < 0.01). In addition, the content of potentially beneficial metabolites (Biotin, beta-Tocotrienol, 7-Chloro-L-tryptophan and Dihydrozeatin) was significantly increased in the AP group (*p* < 0.05). These results indicate that dietary AMPs can significantly improve the body weights, liver antioxidant capacity and jejunum and ileum VH/CD of squabs.

## 1. Introduction

Squab has become a favored source of high-quality meat for consumers due to its high protein and low fat content, as well as its considerable economic value [1]. However, the current squab farming industry still faces major challenges, including low breeding efficiency and frequent outbreaks of intestinal diseases. In traditional farming practices, antibiotics are commonly used to promote growth and prevent diseases. However, the overuse of antibiotics has led to increased bacterial resistance and food safety concerns, which hinder the sustainable development of the industry. With the global implementation of antibiotic restriction policies, the search for safe and effective alternatives to antibiotics has become an urgent priority for sustainable livestock development [2].

Antimicrobial peptides (AMPs) are small polypeptides naturally produced by bacteria, plants, insects, and mammals in response to pathogenic infections. They possess broad-spectrum antimicrobial activity, strong antibacterial efficacy, structural diversity, and good stability, making them promising candidates as green feed additives. Unlike conventional antibiotics, AMPs exert innate inhibitory effects against bacteria, fungi, and viruses without inducing resistance [3,4]. Research has demonstrated that AMPs can enhance intestinal health, boost immune responses, and thereby improve animal production performance [5]. However, most current studies on the mechanisms of AMPs focus on single-index analyses, which limit the comprehensive understanding of their impact on the intestinal microecosystem and metabolic regulatory networks in squabs. Metagenomics enables the identification of the composition and functional profiles of the gut microbiota at the genetic level, offering insights into how AMPs regulate microbial community structure and metabolic activity [6]. Meanwhile, mass spectrometry-based non-targeted metabolomics allows for qualitative and quantitative analysis of metabolites, facilitating the identification of metabolic differences and the discovery of potential biomarkers [7]. The integration of these two approaches provides a powerful strategy to explore the molecular mechanisms underlying the effects of AMPs on squab growth performance from a holistic microbe–host-metabolism interaction perspective.

As the largest digestive, absorptive, and immune organ in the animal body, the intestine plays a critical role in determining growth performance and disease resistance. The composition and stability of the intestinal microbiota are essential for maintaining intestinal health. Dysbiosis of the gut microbiota can significantly impair poultry productivity and increase disease susceptibility [8]. Currently, research on the application of AMPs in squab farming remains in its early stages, with limited studies employing multi-omics approaches to investigate their mechanisms of action. In this study, we hypothesize that adding 200 mg/kg AMPs to the diet will alter the abundance of key microorganisms, further affect metabolic pathways and metabolite levels, and thereby improve the growth performance of squabs. To test this hypothesis, we employed metagenomics and LC-MS non-targeted metabolomics techniques to systematically investigate the effects of AMPs on the growth performance, intestinal microbiota structure, and metabolic profile of squabs. The results of our study aim to provide a theoretical basis for the scientific application of AMPs in squab farming, which holds significant theoretical significance and practical value for promoting the green and efficient development of the squab breeding industry.

## 2. Materials and Methods

### 2.1. Statement of Ethics

All experimental protocols in this study were approved by the Animal Ethics Committee of Xinjiang Agricultural University (approval number: 2023008).

### 2.2. Additives

The AMPs used in the experiment, referred to as Yingtai Double Antibiotic, were provided by Linzhou Zhongnong Yingtai Biological Co., Ltd. (Linzhou, Henan, China). This formulation primarily consists of three components: *Bacillus subtilis* 37-peptide, metabolite No. 3 peptide, and metabolite No. 4 peptide. Specifically, *Bacillus subtilis* 37-peptide comprises 37 amino acid residues and has a molecular weight of 3879.8 daltons; metabolite No. 3 peptide consists of 28 amino acid residues with a molecular weight of 3300 daltons; and metabolite No. 4 peptide contains 9 amino acid residues, with a molecular weight of 1000 daltons.

### 2.3. Experimental Design and Animals

The experimental pigeons and the test site were provided by Lanhai Pigeon Industry Development Co., Ltd., Moyu County, Hetian Region, Xinjiang, China. A total of 120 pairs of healthy adult White Carneau pigeons (2 years old) with consistent egg-laying performance were selected and randomly divided into 2 groups with 10 replicates per group and 6 pairs of breeding pigeons per replicate. All pigeons were fed a basal diet (Table 1) and grit (Table 2) that met the nutritional requirements of meat pigeons throughout the experiment. The ratio of raw grains to pellets in the basal diet was 6:4. AMPs were supplemented into the diet at the manufacturer’s recommended concentration. The treatment groups were as follows: control group (CK, basal diet) and antimicrobial peptide group (AP, basal diet + 200 mg/kg antimicrobial peptides). The experimental pigeons were housed in a three-tiered cage system, with one pair of breeding pigeons per cage. Fertilized eggs were naturally incubated by the parent pigeons, and the resulting squabs were fed by the parents. Each pair of parent pigeons raised two squabs (2 + 2 rearing model). The experimental period lasted 53 days, consisting of a 7-day pre-feeding period (before egg laying), an 18-day incubation period, and a 28-day feeding period (formal experimental phase). Throughout the trial, breeding pigeons had ad libitum access to feed, grit, and water. The pigeon house was illuminated for 16 h daily, the light intensity was set at 20 Lux, with the light source placed 1 m away from the pigeon cages, and natural ventilation was adopted in the pigeon house. All other management practices followed standard protocols. Squabs were weighed using an electronic scale after an overnight fast at 0 and 28 days of age, and feed consumption was recorded. Parameters including average body weight, average daily weight gain, average daily feed intake, and feed conversion ratio per nest were calculated.

### 2.4. Sample Collection

At 28 days of age, ten pigeons were randomly selected from each group, euthanized, and then sampled. Tissue samples from the duodenum, jejunum, and ileum were rinsed with PBS buffer and immediately fixed in 4% paraformaldehyde solution for histological examination of the small intestine. Duodenal contents were collected and stored at −80 °C for enzyme activity analysis. Jejunal and liver tissues were collected and stored at −80 °C for assessment of antioxidant capacity. Ileal contents were collected and stored at −80 °C for analysis of the intestinal microbiota, metabolites, and metabolic pathways.

### 2.5. Antioxidant Capacity

The total antioxidant capacity (T-AOC), total superoxide dismutase (T-SOD), glutathione peroxidase (GSH-Px) activity, and malondialdehyde (MDA) content were measured according to the protocols provided by the assay kits (Nanjing Jiancheng Bioengineering Institute, Nanjing, China).

### 2.6. Digestive Enzyme Activity

The activities of digestive enzymes, including amylase, trypsin, pepsin, and lipase, were determined. The specific operational procedures followed the instructions provided with the assay kits (Nanjing Jiancheng Bioengineering Institute, Nanjing, China).

### 2.7. Intestinal Morphology Assays

Small intestinal tissues were excised from 4% paraformaldehyde fixative, trimmed, dehydrated, and embedded. Serial sections were then prepared using a microtome at a thickness of 5 μm. The sections were stained with hematoxylin and eosin (HE), mounted with neutral balsam, and examined under an optical microscope for imaging. Villus height (VH) and crypt depth (CD) were measured using ImageJ software (version 1.54p, National Institutes of Health, Bethesda, MD, USA), and the (VH/CD) was subsequently calculated.

### 2.8. Analysis of Gut Microbiota

Sampling 1 μg of genomic DNA, the sample is randomly fragmented into segments of approximately 350 bp using a Covaris ultrasonic disruptor to construct the library. The entire library preparation is completed through steps including end repair, addition of A-tails, ligation of sequencing adapters, purification, and PCR amplification. After library construction, the integrity of the library fragments and the size of the inserted fragments are assessed using AATI analysis. If the insert size meets expectations, the accurate concentration of the effective library is quantified using Q-PCR (effective library concentration >3 nM) to ensure the library quality. After the library passes the quality check, different libraries are pooled according to their effective concentrations and target data output requirements, and then subjected to PE150 sequencing.

Fastp (version 0.23.4, HaploX Corp., Shenzhen, China) is used for preprocessing raw data from the sequencing platform to obtain clean data for subsequent analysis. Considering the possibility of host contamination in samples, clean data needs to be blasted to the host database to filter out reads that may come from host origin. Bowtie2 software (version 2.5.4, Johns Hopkins University, Baltimore, MD, USA) is used by default. MEGAHIT software (version 1.2.9, The University of Hong Kong-Beijing Genomics institution, Hong Kong, China) is used for assembly analysis of clean data, and scaftigs without N is obtained by breaking the resulted scaffolds from the N junction.

With the default parameters, MetaGeneMark (Georgia Institute of Technology, Atlanta, GA, USA) is used to perform ORF prediction for scaftigs (≥500 bp) of each sample, and the information with a length less than 100 nt in the prediction results is filtered out. For the ORF prediction results, CD-HIT software (version 4.9.1, Burnham Institute, La Jolla, CA, USA) is used to eliminate redundancy and obtain the non-redundant initial gene catalogue (the nucleic acid sequences encoded by successive non-redundant genes are called genes). Clean data of each sample is aligned to the initial gene catalogue by using Bowtie2 to calculate the number of reads of the genes on each sample alignment. Genes with reads <= 2 in each sample are filtered out to finally determine the gene catalogue for subsequent analysis.

DIAMOND software (version 2.1.9, Max Planck Institute for Developmental Biology, Tübingen, Germany) is used for alignment of unigenes sequences with the Micro_NR database, which includes sequences from bacteria, fungi, archaea, and viruses extracted from NCBI’s NR database (https://www.ncbi.nlm.nih.gov/, accessed on 13 May 2025). From the alignment results of each sequence, the one with evalue <= min. evalue ×10 is selected. Since each sequence may have multiple alignment results, the LCA algorithm (applied to systematic taxonomy of MEGAN software (version 6.25.10, University of Tuebingen, Tübingen, Germany) is adopted to determine the species annotation information of the sequence. Principal component analysis (PCA) was performed using R software (Version 4.0.3, The University of Auckland, Auckland, New Zealand), followed by the application of LEfSe software (version 1.1.2, Harvard University, Cambridge, MA, USA) to identify differentially abundant species between groups based on an LDA Score threshold of ≥3.

DIAMOND software (version 2.1.9, Max Planck Institute for Developmental Biology, Tübingen, Germany) is used to align unigenes with those in the functional database. Functional databases include the KEGG database (http://www.kegg.jp/kegg/, accessed on 13 May 2025), eggNOG database (http://eggnogdb.embl.de/#/app/home, accessed on 13 May 2025). From the alignment results of each sequence, the best blast hit results are selected for subsequent analysis. The sequencing and data analysis were carried out with technical support from Novogene Co., Ltd. (Beijing, China).

### 2.9. Metabolomics Analysis

The samples (100 μL) were placed in the EP tubes and resuspended with prechilled 80% methanol by well vortex. Then the samples were incubated on ice for 5 min and centrifuged at 15,000× *g*, 4 °C for 20 min. Some of the supernatant was diluted to a final concentration containing 53% methanol by LC-MS grade water. The samples were subsequently transferred to a fresh Eppendorf tube and then were centrifuged at 15,000× *g*, 4 °C for 20 min. Finally, the supernatant was injected into the LC-MS/MS system analysis.

UHPLC-MS/MS analyses were performed using a Vanquish UHPLC system (Thermo Fisher, Germering, Germany) coupled with an Orbitrap Q Exactive TM HF mass spectrometer or Orbitrap Q Exactive TMHF-X mass spectrometer (Thermo Fisher, Germering, Germany) in Novogene Co., Ltd. (Beijing, China). Samples were injected onto a Hypersil Goldcolumn (100 × 2.1 mm, 1.9 μm) using a 12-min linear gradient at a flow rate of 0.2 mL/min. The eluents for the positive and negative polarity modes were eluent A (0.1% FA in Water) and eluent B (Methanol). The solvent gradient was set as follows: 2% B, 1.5 min; 2–85% B, 3 min; 85–100% B, 10 min; 100–2% B, 10.1 min; 2% B, 12 min. A Q Exactive TM HF mass spectrometer was operated in positive/negative polarity mode with spray voltage of 3.5 kV, capillary temperature of 320 °C, sheath gas flow rate of 35 psi and aux gas flow rate of 10 L/min, S-lens RF level of 60, and Aux gas heater temperature of 350 °C.

### 2.10. Data Statistics and Analysis

The original data were organized and analyzed using Excel (Microsoft office 2019, Microsoft Corp., Redmond, WA, USA). Statistical analysis was performed using an independent samples *t*-test with SPSS software (version 27.0, IBM Corp., Armonk, NY, USA). Differences were considered statistically significant when *p* < 0.05 and non-significant when *p* > 0.05. Graphs were generated using GraphPad Prism (version 8.0.2, GraphPad Software Corp., San Diego, CA, USA).

Quality control of QC samples was performed using R software (version 3.4.3, The University of Auckland, Auckland, New Zealand). PCA and partial least squares discriminant analysis (PLS-DA) were performed with metaX (version 1.4.2, Beijing Genomics institution, Shenzhen, China). The metabolites with VIP > 1 and *p*-value < 0.05 and fold change ≥1.2 or FC ≤ 0.833 were considered to be differential metabolites. Volcano plots and bubble plots were generated using the ggplot2 package in R language. The functions of these metabolites and metabolic pathways were studied using the KEGG database. The metabolic pathway enrichment of differential metabolites was performed: when the ratio was satisfied by x/n > y/N, a metabolic pathway was considered enriched; when the *p*-value of a metabolic pathway was <0.05, the metabolic pathway was considered statistically significantly enriched. Metabolic correlations were analyzed using the Pearson correlation coefficient method.

## 3. Results

### 3.1. Growth Performance

**Figure 1:**
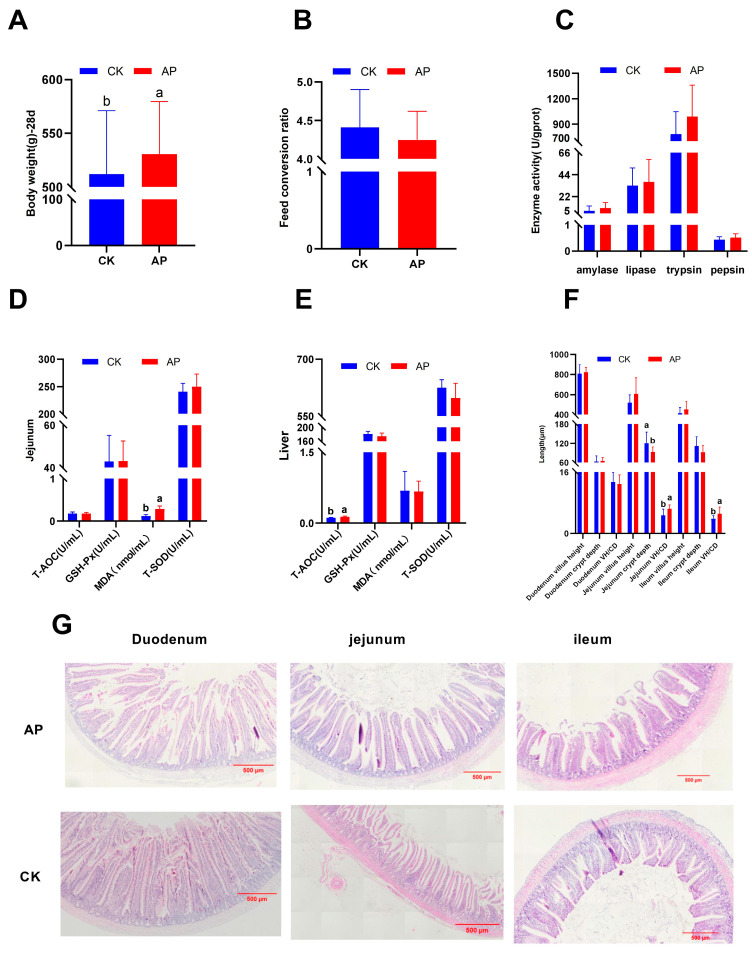
Effects of dietary AMPs on growth performance, enzyme activity, antioxidant capacity, and intestinal tissue morphology of squabs. (**A**,**B**) Body weight and feed conversion ratio at 28 days of age. (**C**) Duodenal amylase, lipase, trypsin, and pepsin activities. (**D**,**E**) Oxidation and antioxidant indicators of the jejunum and liver. (**F**,**G**) Histology of duodenum, jejunum, and ileum (HE staining, 40×). CK was the control group, and AP was the antimicrobial peptide group. Bar graphs marked with different letters a and b indicate significant differences (*p* < 0.05).

As illustrated in Figure 1A,B, the body weight of 28-day-old squabs in the AP group was significantly higher than that in the control group (*p* < 0.05). In contrast, the feed conversion ratio from 0 to 28 days of age did not differ significantly between groups, although a decreasing trend was observed (*p* > 0.05).

### 3.2. Enzymatic Activity

As illustrated in Figure 1C, dietary supplementation with AMPs did not significantly affect the activities of amylase, lipase, trypsin, or pepsin in the duodenum of squabs (*p* > 0.05).

### 3.3. Antioxidant Capacity

As illustrated in Figure 1D,E, compared with the control group, the AP group exhibited a significant increase in MDA content in the jejunum (*p* < 0.05) and a significant improvement in liver T-AOC (*p* < 0.05), with no statistically significant differences observed in other measured parameters (*p* > 0.05).

### 3.4. Intestinal Morphology

As illustrated in Figure 1F, compared with the control group, the AP group exhibited a significant reduction in jejunal CD and a marked increase in the VH/CD (*p* < 0.05). Similarly, a significant increase in the VH/CD was observed in the ileum (*p* < 0.05), while no statistically significant differences were found in other measured parameters (*p* > 0.05). Histological analysis revealed that the intestinal villi in the duodenum, jejunum, and ileum of the AP group were relatively intact, well-organized, and exhibited minimal histological damage. In contrast, the intestinal villi in the CK group showed compromised integrity, disorganized arrangement, and varying degrees of histological injury (Figure 1G).

### 3.5. Analysis of Gut Microbiota

#### 3.5.1. Metagenomic Sequencing Information

A total of 144.97 G of sequencing data was generated from 20 ileal content samples of squabs, with an average of 7.25 G per sample. Following quality control and host contamination removal, 136.76 G of high-quality sequences were retained, averaging 6.84 G per sample. The Q20 and Q30 values of the effective sequences exceeded 96.64% and 91.28%, respectively, while the GC content ranged from 38.71% to 51.92%, indicating high sequencing accuracy and suitability for downstream analysis (Table 3). After assembly, a total of 7,193,094 Scaftigs were obtained, with an average of 359,654.7 per sample. The average N50 and N90 values were 1927.5 bp and 651.55 bp, respectively, suggesting satisfactory assembly performance (Table 4).

#### 3.5.2. Alpha Diversity Analysis

**Figure 2:**
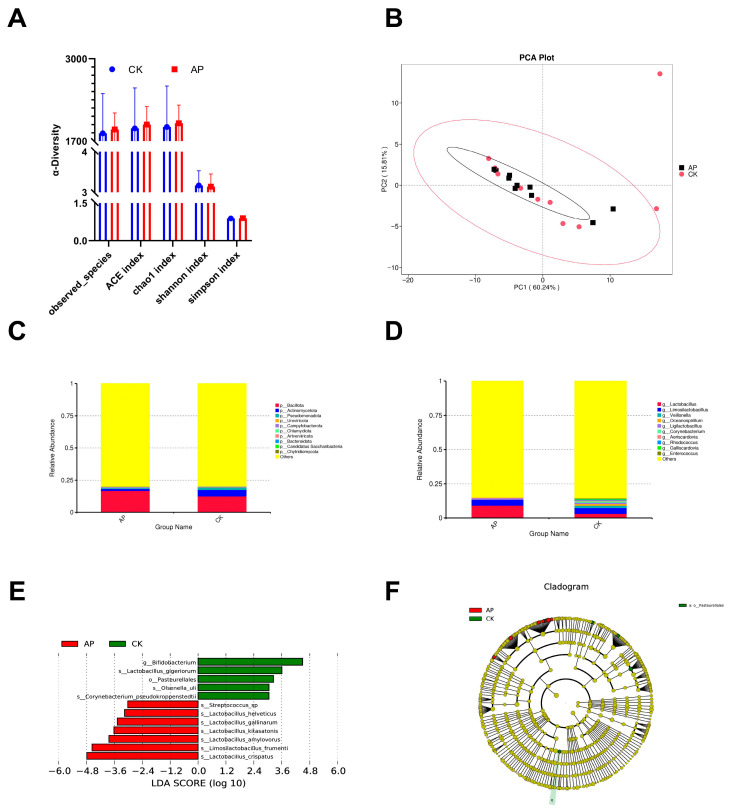
The effects of dietary antimicrobial peptide supplementation on the ileal microbiota of squabs. (**A**) Alpha diversity indices of the intestinal microbiota, including ACE, Chao1, observed species, Shannon, and Simpson indices. (**B**) Principal component analysis (PCA) illustrating structural differences in the intestinal microbiota between the control group (CK) and the antimicrobial peptide group (AP). (**C**) Bar charts depicting the relative abundance of microbial phyla across groups. (**D**) Bar charts depicting the relative abundance of microbial genera across groups. (**E**) Bar chart showing the distribution of LDA scores for differentially abundant taxa (LDA > 3). (**F**) Phylogenetic representation of differentially abundant taxa. Circles radiating from the center outward represent taxonomic levels from phylum to species. Each small circle represents a taxon at that classification level, with circle diameter proportional to relative abundance. Coloring scheme: taxa without significant differences are colored yellow, while biomarkers are colored according to their group association. Red nodes indicate taxa enriched in the AP group, and green nodes indicate taxa enriched in the CK group.

To evaluate the regulatory effects of AMPs on microbial diversity, alpha diversity analysis of the ileal contents of squabs was conducted (Figure 2A). Following the administration of a diet supplemented with AMPs, no statistically significant differences were observed across all diversity indices (including observed_species, ACE, Chao1, Shannon, and Simpson) (*p* > 0.05).

#### 3.5.3. β Diversity Analysis

PCA was applied to reduce the dimensionality of the multivariate dataset. PC1 and PC2 accounted for 60.24% and 15.81% of the variance in the samples, respectively. In the coordinate space, samples from the AP group and the CK group exhibited both separation and overlap, indicating that the microbial community structures of the two groups shared certain similarities while also displaying distinct differences (Figure 2B).

#### 3.5.4. Analysis of the Difference in Intestinal Microflora Between the AP Group and CK Group

We investigated the effects of AMPs on the intestinal microbiota structure in squabs using metagenomic sequencing. Based on the relative abundance tables at the phylum and genus levels, we selected the top 10 most abundant taxa in each group and categorized the remaining taxa as “Others”. Relative abundance bar charts at both phylum and genus levels were then generated for each sample. At the phylum level, the top 10 phyla identified in the ileal contents of both groups included *Bacillota*, *Actinomycetota*, *Pseudomonadota*, *Uroviricota*, *Campylobacterota*, *Chlamydiota*, *Artverviricota*, *Bacteroidota*, *Candidatus Saccharibacteria*, and *Chytridiomycota*. Among these, *Bacillota* exhibited the highest relative abundance (AP group: 16.59%; CK group: 12.39%) and was identified as the dominant phylum. Notably, the relative abundance of *Bacillota* was higher in the AP group compared to the CK group, although the difference was not statistically significant (*p* > 0.05) (Figure 2C). At the genus level, the top 10 genera included *Lactobacillus*, *Limosilactobacillus*, *Veillonella*, *Oceanospirillum*, *Ligilactobacillus*, *Corynebacterium*, *Aeriscardovia*, *Rhodococcus*, *Galliscardovia*, and *Enterococcus*. *Lactobacillus* was the most abundant genus (AP group: 9.07%; CK group: 3.07%) and was considered the dominant genus. Similar to Bacillota, the relative abundance of *Lactobacillus* was higher in the AP group than in the CK group, but again, the difference did not reach statistical significance (*p* > 0.05) (Figure 2D).

To identify potential biomarkers with significant intergroup differences, we performed LEfSe analysis to detect alterations in the ileal microbiota following AMP supplementation (Figure 2E,F). The results revealed 12 taxa that showed statistically significant differences between the two groups (*p* < 0.05). Among these, five taxa were enriched in the CK group: *Bifidobacterium*, *Lactobacillus_gigeriorum*, *Pasteurellales*, *Olsenella_uli*, and *Corynebacterium_pseudokroppenstedtii*. Seven taxa were enriched in the AP group: *Streptococcus sp.*, *Lactobacillus helveticus*, *Lactobacillus gallinarum*, *Lactobacillus kitasatonis*, *Lactobacillus amylovorus*, *Limosilactobacillus frumenti*, and *Lactobacillus crispatus*. Notably, *Lactobacillus* species constituted the core microbial group in the AP group.

#### 3.5.5. Functional Annotation of Gut Microbiota in Squabs

**Figure 3:**
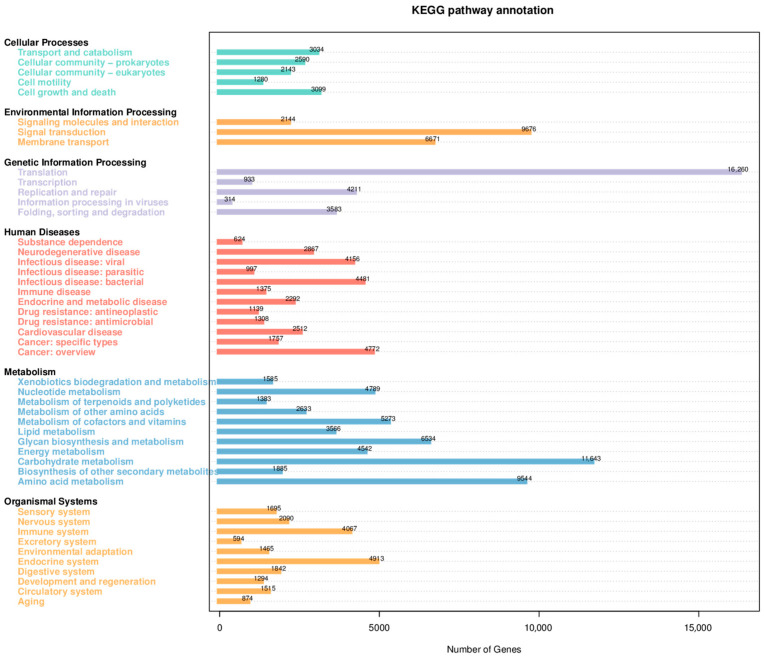
Statistical diagram of the number of annotated genes in the KEGG database.

Functional annotation of the differential microbiota between the AP group and the CK group was carried out using the KEGG database, with results shown in Figure 3. The differentially abundant microbial taxa were predominantly enriched in functional pathways including Cellular Processes, Environmental Information Processing, Genetic Information Processing, Human Diseases, Metabolism, and Organismal Systems. Among these, the pathway of Translation under Genetic Information Processing exhibited the highest abundance. Additionally, signal transduction and membrane transport within Environmental Information Processing, as well as carbohydrate metabolism, amino acid metabolism, and glycan biosynthesis and metabolism under the Metabolism category, were biological processes with substantial enrichment of differential microbiota between the two groups.

**Figure 4:**
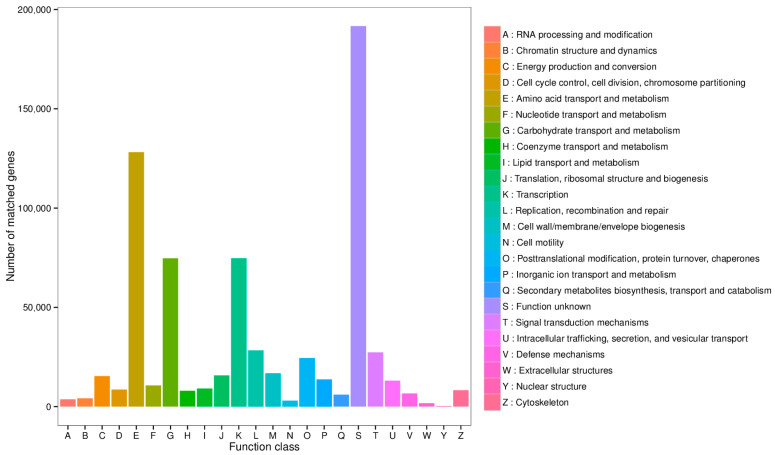
Statistical diagram of the number of annotated genes in the EggNOG database.

Functional annotation was further performed using the eggNOG database, with results displayed in Figure 4. Excluding genes with function unknown, the most abundant functional category was amino acid transport and metabolism, followed by carbohydrate transport and metabolism, and transcription.

### 3.6. Effects of Dietary Antimicrobial Peptides on Ileum Metabolomics of Squabs

#### 3.6.1. Sample Quality Control (QC) Analysis

**Figure 5:**
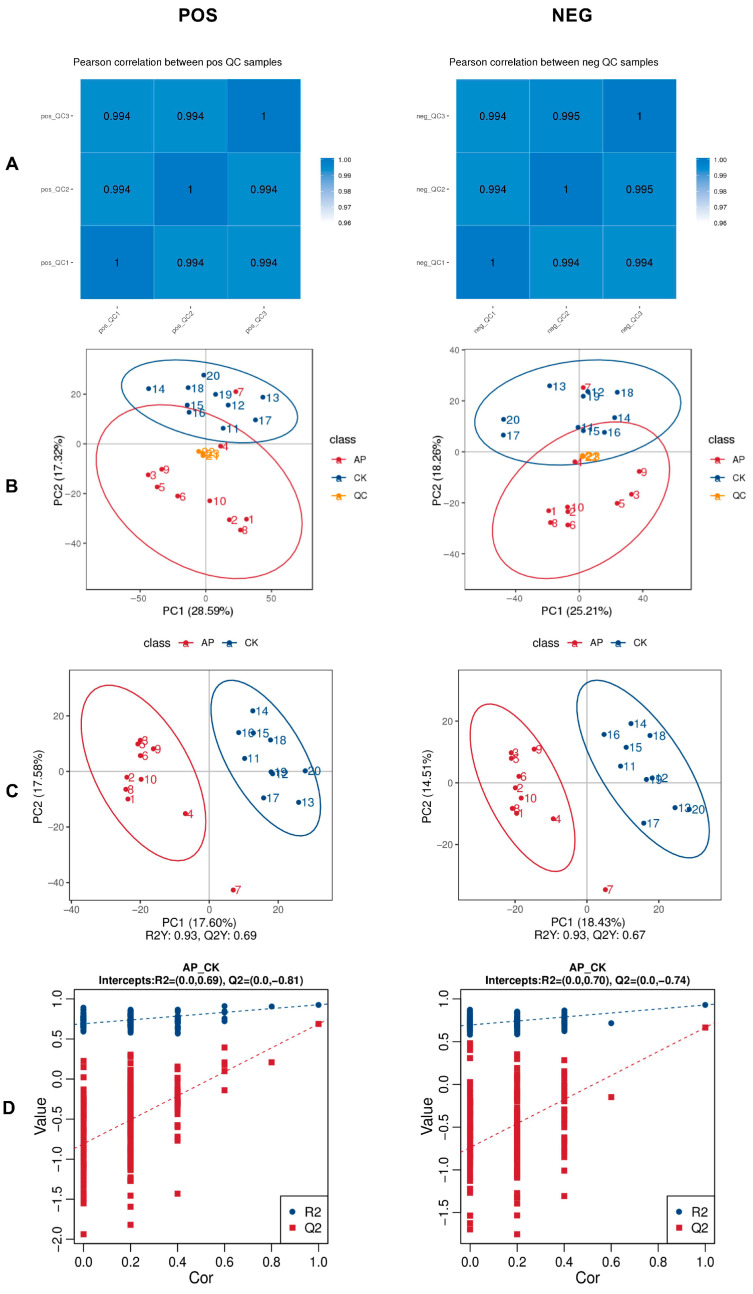
Metabolomic features of each sample. (**A**) Correlation analysis of QC samples. (**B**) Principal component analysis (PCA) of the total sample. (**C**) Plots of PLS scores for samples of intestinal contents in the CK and AP groups. (**D**) Permutation test plot of PLS-DA analysis model. The positive ion mode (POS) is shown on the left and the negative ion mode (NEG) is shown on the right.

The Pearson correlation coefficients among QC samples were calculated based on the relative quantitative values of metabolites. All correlation coefficients exceeded 0.99, indicating a highly stable detection process and reliable data quality (Figure 5A). PCA was performed on all peaks extracted from both experimental and QC samples. The tight clustering of QC samples in the PCA plot further confirms the methodological stability and high accuracy of metabolite identification (Figure 5B).

#### 3.6.2. Partial Least Squares Discriminant Analysis (PLS-DA)

In this study, the PLS-DA positive ion model exhibited an R2Y value of 0.93 and a Q2Y value of 0.69, while the negative ion model showed similar performance with R2Y = 0.93 and Q2Y = 0.67. Both R2Y and Q2Y exceeded 0.5, and the R2Y values were close to 1, indicating that the models possessed strong explanatory power and predictive accuracy, and were therefore considered stable and reliable. Clear separation between the CK and AP groups was observed in both ionization modes, demonstrating effective discrimination between the two groups (Figure 5C). To assess model robustness and rule out overfitting, permutation tests were conducted by randomly shuffling group labels 200 times. The resulting Q2 values for the positive and negative ion models were −0.81 and −0.74, respectively. The intercepts of the Q2 regression lines with the Y-axis were both below zero, and R2 values were consistently higher than Q2 values. These results confirm the absence of overfitting and support the conclusion that the models accurately represent the data, making them suitable for subsequent differential metabolite screening (Figure 5D).

#### 3.6.3. Differential Metabolite Analysis

**Figure 6:**
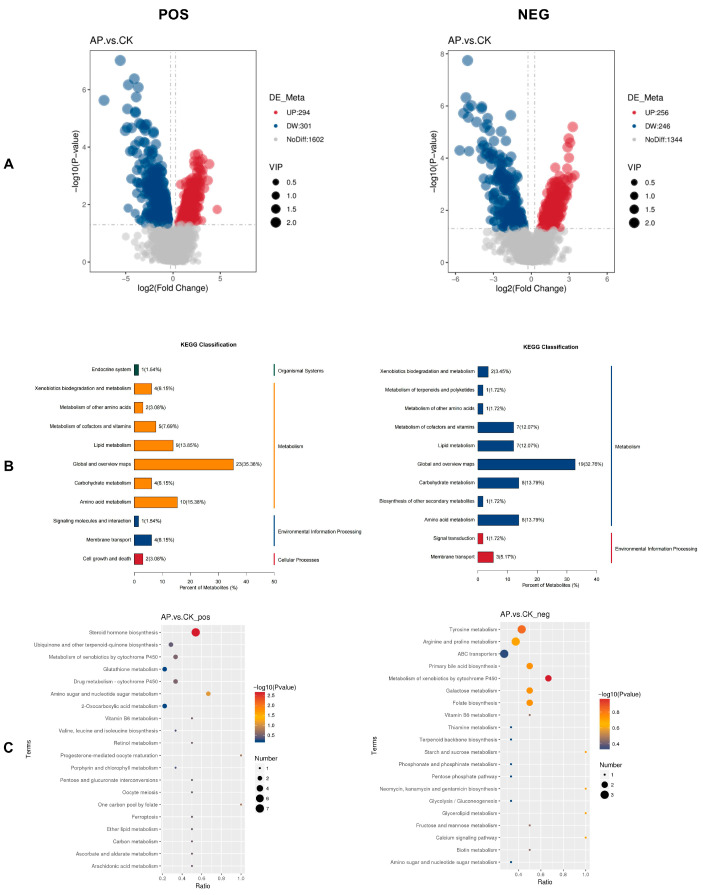
Results of differential metabolite analysis in the intestines of the CK group and AP group. (**A**) Volcano plot of differential metabolites. (**B**) KEGG classification map of differential metabolites. (**C**) Pathway enrichment analysis bubble map of differential metabolites. The larger the Ratio value, the higher the enrichment degree of differential metabolites in the pathway. The color of the dots represents the *p*-value of the hypergeometric test, and the size of the dots represents the number of differential metabolites in the corresponding pathway. The positive ion mode (POS) is shown on the left, and the negative ion mode (NEG) is shown on the right.

Using non-targeted metabolomics technology (LC-MS/MS), a total of 2197 metabolites in positive ion mode and 1846 metabolites in negative ion mode were identified. Of these, 595 metabolites in positive ion mode exhibited significant differences, including 294 upregulated and 301 downregulated metabolites. Similarly, 502 metabolites in negative ion mode showed significant variation, with 256 upregulated and 246 downregulated metabolites (Figure 6A).

#### 3.6.4. KEGG Classification and Enrichment Analysis

Statistical analysis was performed on the annotated differentially expressed metabolites based on KEGG database-derived classification data (Figure 6B). In positive ion mode (POS), these metabolites were categorized into four functional classes: Organismal Systems, Metabolism, Environmental Information Processing, and Cellular Processes. In negative ion mode (NEG), the metabolites were grouped into two categories: Metabolism and Environmental Information Processing. Notably, the Metabolism category exhibited the highest proportion in both ionization modes.

To further explore the potential biological functions of these annotated differentially expressed metabolites in the AP and CK groups, KEGG functional enrichment analysis was carried out. Figure 6C presents the top 20 enriched KEGG pathways, which were predominantly associated with steroid hormone biosynthesis, tyrosine metabolism, arginine and proline metabolism, ABC transporters, primary bile acid biosynthesis, metabolism of xenobiotics by cytochrome P450, galactose metabolism, and folate biosynthesis. Among these pathways, steroid hormone biosynthesis showed the most significant difference between the two groups (*p* < 0.01).

#### 3.6.5. Screening of Marker Metabolites

**Figure 7:**
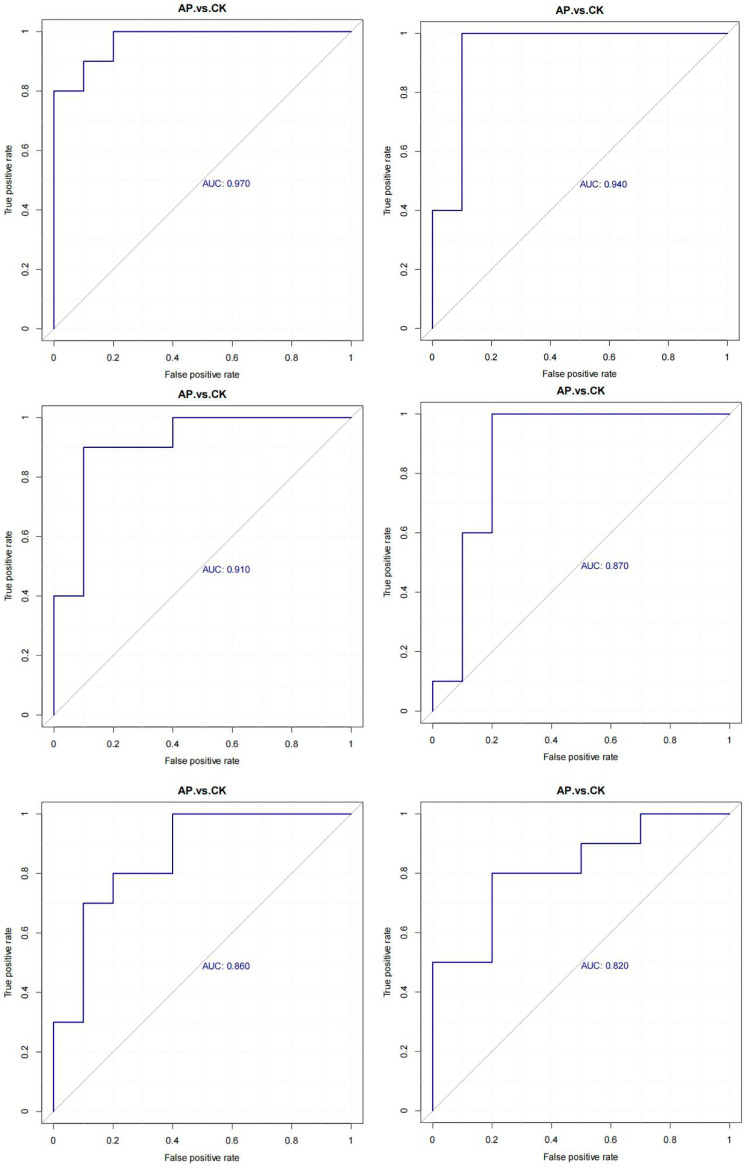
ROC curves of differential metabolites.

Receiver operating characteristic (ROC) curve analysis was employed to evaluate the predictive performance of each potential marker metabolite based on the identified metabolic differences. The area under the curve (AUC) reflects the accuracy of prediction, with values closer to 1 indicating higher predictive accuracy. Specifically, AUC values between 0.5 and 0.7 suggest low predictive accuracy, those between 0.7 and 0.9 indicate moderate predictive accuracy, and values above 0.9 reflect high predictive accuracy. An AUC of 0.5 indicates no predictive value for the biomarker. Among the metabolites analyzed, Biotin (AUC = 0.970), beta-Tocotrienol (AUC = 0.940), 7-Chloro-L-tryptophan (AUC = 0.910), Dihydrozeatin (AUC = 0.870), Progesterone (AUC = 0.860), and 17β-Estradiol (AUC = 0.820) all exhibited AUC values greater than 0.80, demonstrating strong predictive performance and supporting their selection as potential marker metabolites (Figure 7).

### 3.7. Correlation Analysis

**Figure 8:**
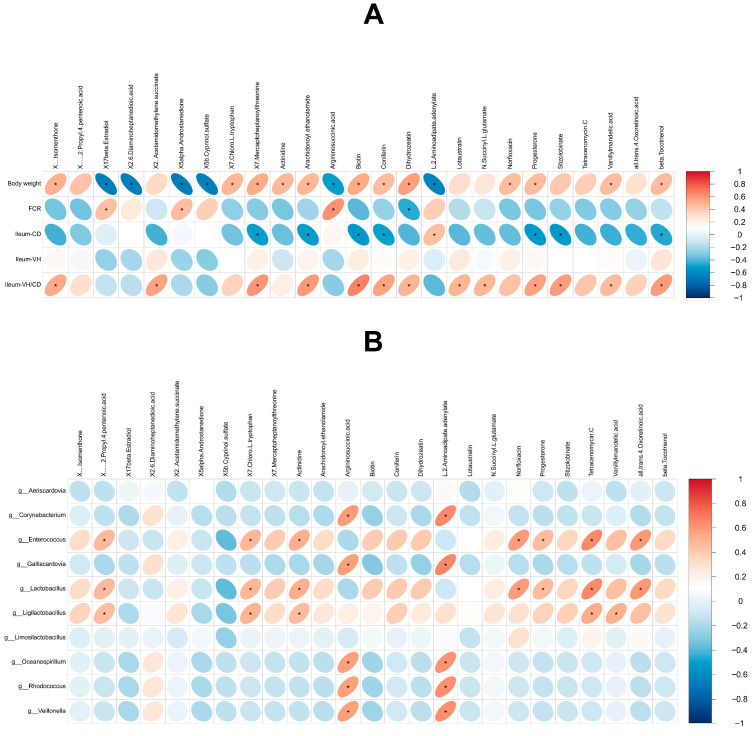
Results of correlation analysis. (**A**) Correlation analysis between phenotypic data and metabolites. (**B**) Correlation analysis between microbiota and metabolites. Red indicates positive correlation and blue indicates negative correlation. “*” indicates a significant correlation (*p* < 0.05).

A correlation analysis was performed between phenotypic data of 28-day-old squabs, including body weight and ileum villus height/crypt depth (ileum-VH/CD), and key differential metabolites (Figure 8A). The results revealed that body weight was significantly positively correlated with (+)-Isomenthone, 7-Chloro-L-tryptophan, 7-Mercaptoheptanoylthreonine, Actinidine, Arachidonoyl ethanolamide, Biotin, Coniferin, Dihydrozeatin, Norfloxacin, Progesterone, Vanillylmandelic acid, and beta-Tocotrienol (*p* < 0.05). In contrast, it was significantly negatively correlated with 17beta-Estradiol, 2,6-Diaminoheptanedioic acid, 5alpha-Androstanedione, 5b-Cyprinol sulfate, Argininosuccinic acid, and L-2-Aminoadipate adenylate (*p* < 0.05). Ileum-VH/CD showed significant positive correlations with (+)-Isomenthone, 2-(Acetamidomethylene)succinate, 7-Mercaptoheptanoylthreonine, Arachidonoyl ethanolamide, Biotin, Coniferin, Dihydrozeatin, Lotaustralin, N-Succinyl-L-glutamate, Progesterone, Stizolobinate, Vanillylmandelic acid, and beta-Tocotrienol (*p* < 0.05).

Furthermore, a correlation analysis was conducted between the top 10 intestinal bacterial genera and key differential metabolites to explore potential interactions between gut microbiota and metabolites (Figure 8B). Notably, compared with the CK group, the relative abundance of *Lactobacillus*, a known probiotic genus, in the AP group was significantly positively correlated with several upregulated metabolites, including (+/−)-2-Propyl-4-pentenoic acid, 7-Chloro-L-tryptophan, Actinidine, Norfloxacin, Progesterone, Tetracenomycin C, and all-trans-4-Oxoretinoic acid (*p* < 0.05).

## 4. Discussion

The growth cycle of squabs is relatively short, and their ability to digest and absorb nutrients directly influences their growth and development. In recent years, antimicrobial peptides (AMPs), as eco-friendly and effective alternatives to antibiotics, have been increasingly used as feed additives in livestock and poultry production to enhance animal health and productivity. In this study, dietary supplementation with 200 mg/kg of AMPs significantly improved the growth performance of squabs, particularly increasing their body weight at 28 days of age. This finding aligns with previous studies [9,10]. The growth-promoting effects of AMPs may be attributed to their capacity to modulate the intestinal microbiota by suppressing harmful bacteria and promoting the proliferation of beneficial microbes, thereby enhancing intestinal nutrient digestion and absorption efficiency [2]. Notably, these effects may also be partially mediated through immunomodulatory functions [11]. AMPs can strengthen the intestinal mucosal immune barrier, reduce pathogen invasion, and mitigate energy loss caused by inflammatory responses, thereby redirecting more nutrients toward growth and development [12]. Future studies should investigate the long-term effects of varying AMP dosages to provide more comprehensive data for optimizing efficient and healthy breeding strategies for squabs.

Antioxidant indices serve as critical indicators for evaluating oxidative stress status and the body’s antioxidant capacity. Total antioxidant capacity (T-AOC) reflects the overall antioxidant potential of all endogenous antioxidants. Total superoxide dismutase (T-SOD) indicates the capacity to scavenge superoxide anions, while glutathione peroxidase (GSH-Px) reflects the ability to neutralize peroxides and repair lipid peroxidation damage. Malondialdehyde (MDA), a terminal product of lipid peroxidation, is commonly used as a biomarker of oxidative stress and cellular damage [13,14,15,16]. Wang et al. [17] demonstrated that supplementing the basal diet of Ross 308 broilers with 100 or 200 mg/kg of the antimicrobial peptide Gal-13 significantly increased liver and serum GSH-Px activity while reducing MDA levels. Similarly, Cao et al. [18] reported that adding 200 mg/kg of the antimicrobial peptide microcin J25 to the diet of American King Pigeons significantly enhanced GSH-Px and SOD activities in both the liver and intestine, thereby improving systemic antioxidant capacity. In this study, dietary supplementation with 200 mg/kg of AMPs significantly increased liver T-AOC in squabs but also led to a significant elevation in jejunal MDA levels. This suggests that while AMPs enhance antioxidant defenses, they may also induce a certain degree of oxidative stress, which contrasts with previous findings. The liver, as a central organ for detoxification and metabolism, may benefit from AMP-induced activation of antioxidant enzyme expression and activity, thereby improving the clearance of reactive oxygen species (ROS) and overall antioxidant capacity [19]. However, the observed increase in jejunal MDA levels may result from the rapid modulation of intestinal microecology and mucosal architecture by AMPs. Specifically, AMPs may alter gut microbiota composition by suppressing pathogenic bacteria while stimulating the metabolic activity of beneficial microbes, potentially causing a transient redox imbalance and increased ROS production [20]. Additionally, AMP-induced acceleration of intestinal cell proliferation and metabolism may also contribute to elevated ROS levels [21]. This localized oxidative stress may represent a transitional adaptation phase of the gut to AMP intervention, which did not negatively affect growth performance or intestinal morphology. Whether this condition can be resolved through activation of the intestinal antioxidant defense system remains to be elucidated. Moreover, the liver and intestine are functionally interconnected in oxidative stress regulation. Enhanced liver antioxidant capacity may supply antioxidant agents to the intestine via systemic circulation, while intestinal oxidative stress may feedback-regulate liver metabolic and detoxification functions [22].

The ileum, as the terminal segment of the small intestine, plays a pivotal role in nutrient digestion, absorption, and transport. Its structural and functional integrity directly affects nutrient utilization efficiency and overall health [23]. Key morphological indicators such as villus height, crypt depth, and the villus-to-crypt ratio (VH/CD) are widely used to assess intestinal health. Increased villus height expands the absorptive surface area, facilitating the transcellular transport of nutrients like glucose, amino acids, and fatty acids. Conversely, increased crypt depth is often associated with impaired nutrient absorption and reduced growth performance. Therefore, a higher villus height and VH/CD, along with a lower crypt depth, generally indicate improved intestinal nutrient absorption capacity [24,25]. Zhang et al. [26] found that supplementing the diet of yellow-feathered broilers with 100 or 200 mg/kg of the antimicrobial peptide Plectasin significantly increased villus height, crypt depth, and VH/CD in the duodenum, jejunum, and ileum at 21 days of age. Similarly, Dai et al. [27] reported that dietary supplementation with Microcin C7 significantly increased intestinal villus height and VH/CD while reducing crypt depth in Arbor Acres broilers. In this study, 200 mg/kg of AMPs significantly improved the VH/CD in both the jejunum and ileum of squabs, consistent with previous findings. From the perspective of host–microbiota interactions, the improvement in VH/CD may be closely linked to alterations in gut microbiota composition. AMPs can selectively inhibit pathogenic bacteria such as *E. coli* and *Salmonella*, while promoting the proliferation of beneficial bacteria like *Bifidobacterium* and *Lactobacillus*, thereby optimizing intestinal microecology. Short-chain fatty acids (SCFAs), particularly butyrate, produced by beneficial microbes, serve as energy substrates for intestinal epithelial cells and promote villus cell proliferation and differentiation [28]. Furthermore, AMPs may regulate the intestinal metabolic profile to enhance the expression of nutrient transporters, thereby improving nutrient absorption efficiency and providing metabolic support for villus growth, which synergistically increases the VH/CD [29].

Intestinal digestive enzymes are essential for nutrient digestion and are considered key indicators of poultry production performance and feed utilization efficiency. Amylase, lipase, and protease work synergistically to sequentially digest carbohydrates, lipids, and proteins, respectively, serving as the primary drivers of nutrient absorption in poultry [30]. Zhu et al. [31] found that supplementing the diet of Arbor Acres broilers with 20 mg/kg of the antimicrobial peptide Mastoparan X significantly upregulated the mRNA expression of intestinal digestive enzyme-related genes such as FABP2 and SLC2A5/GLUT5, thereby improving enzyme activity and intestinal structure and function. In contrast, this study found that dietary supplementation with 200 mg/kg of AMPs had no significant effect on digestive enzyme activity in squabs, which contradicts previous findings. From a mechanistic perspective, digestive enzyme synthesis and secretion are regulated by multiple factors, including the intestinal microenvironment, hormonal levels, and nutritional status [32]. The digestive enzyme activity in squabs may primarily depend on their developmental stage. The modulation of gut microbiota and mucosal structure by AMPs may not have exceeded the intrinsic regulatory threshold for digestive enzyme activity, resulting in no observable changes. However, the absence of significant changes in enzyme activity does not imply that AMPs have no impact on digestive function. Integrating findings from other experimental results, it is plausible that AMPs may indirectly enhance nutrient digestion and utilization through non-enzymatic mechanisms, such as optimizing the intestinal absorption environment or regulating metabolic pathways. Future studies should extend the experimental duration, expand the range of digestive enzyme assays, and incorporate transcriptomic analyses to explore the potential regulatory effects of AMPs on digestive enzyme gene expression. This would provide a more comprehensive understanding of the relationship between AMPs and digestive function in squabs, supporting their precise application in pigeon production.

The majority of intestinal microbiota indirectly regulate substance absorption, immune responses, energy metabolism, and signaling pathway activation through bacterial metabolic products and other physiological processes [33]. In this study, the effects of AMPs on the ileal microbiota and metabolic profiles of squabs were further investigated using metagenomic and LC-MS-based untargeted metabolomics approaches. We found that dietary supplementation with AMPs significantly altered the steroid hormone biosynthesis pathway in squabs and modified the levels of multiple metabolites. These alterations provide valuable insights into the underlying mechanisms by which AMPs influence squab growth performance.

Cholesterol serves as a major lipid component of cell membranes and acts as a precursor for steroid hormones, neurosteroids, and bile acids. The biosynthesis of steroid hormones plays a crucial role in maintaining physiological homeostasis and regulating animal growth and development. Changes in steroid hormone metabolites can serve as key biomarkers reflecting animal health and production performance [34]. 17β-Estradiol (E2), the most biologically active estrogen, has been shown to negatively affect the growth and development of vertebrates [35]. Studies have demonstrated that high-dose E2 administration significantly inhibits the growth rate of juvenile tiger pufferfish by interfering with gonadal differentiation and energy allocation, resulting in reduced weight gain and survival rates with increasing E2 dosage [36]. Wang et al. [37] reported that although E2 treatment increased feed intake in bluegill sunfish, it significantly reduced growth rates. Specifically, the final body weights of fish receiving 100 and 150 mg/kg E2 were significantly lower than those of the control group, with even more pronounced growth inhibition observed at 200 mg/kg E2. In this study, metabolomic and Pearson correlation analyses revealed that E2 levels were significantly downregulated in the AP group and exhibited a strong negative correlation with squab body weight. This suggests that reducing E2 content may be beneficial for squab growth and development. It should be noted that the aforementioned inhibitory effects of E2 on growth are mostly derived from fish studies, with potential interspecies differences. Additionally, the E2 level detected in this study is that in the intestine, which may differ from the E2 level reflecting the systemic endocrine status. Future studies could further verify the regulatory role of intestinal E2 through combined detection. Progesterone, as a key steroid hormone, has been shown to enhance energy and nutrient metabolism by modulating intestinal function and influencing the secretion of growth-promoting hormones, thereby significantly improving animal growth performance [38]. In this study, progesterone levels were positively correlated with the body weight of 28-day-old squabs, consistent with previous findings that progesterone promotes muscle protein synthesis via activation of the insulin-like growth factor-1 (IGF-1) signaling pathway [38]. As a key intermediate in the steroid hormone biosynthesis pathway, elevated progesterone levels may influence growth through two potential mechanisms. First, it may enhance the intestinal VH/CD, thereby optimizing nutrient absorption capacity. Second, progesterone was positively correlated with *Lactobacillus* abundance, which can promote intestinal epithelial cell proliferation through the production of short-chain fatty acids (SCFAs), improve gut microecology, and regulate intestinal metabolic profiles, thereby enhancing intestinal health and nutrient absorption and further supporting growth [39]. However, it should be noted that the current research conclusions regarding the growth-promoting effect of progesterone are mostly based on mammals or other avian species. Whether there is interspecific specificity in its role in squabs still requires further in-depth investigation in subsequent studies.

Correlation analysis between metabolites and ileal microbiota revealed a positive association between biotin and *Lactobacillus*. Biotin levels were significantly increased in the AP group and were enriched in the ABC transporter metabolic pathway. ABCG2, also known as breast cancer resistance protein (BCRP), belongs to the ATP-binding cassette (ABC) transporter family and functions as a single-chain transmembrane protein that mediates substrate transport across membranes via ATP hydrolysis. It is predominantly expressed on the apical membranes of epithelial cells in tissues such as the intestine, kidney, liver, placenta, and blood–brain barrier, playing a role in the excretion of endogenous substances (e.g., bilirubin, uric acid, estrogen, biotin) and exogenous compounds (e.g., anticancer drugs, antibiotics) [40]. Studies have shown that human cathelicidin antimicrobial peptide LL-37 and its analogs can bind to ABCG2, inhibit its ATPase activity, and reduce substrate efflux. This inhibition may enhance intestinal biotin uptake and improve its bioavailability [41]. As a cofactor essential for intracellular carboxylase activity and the metabolism of glucose, amino acids, and fatty acids, biotin promotes amino acid conversion and fatty acid synthesis, thereby providing an energy and material basis for animal growth [42], and also exerts anti-inflammatory effects by inhibiting NF-κB activity [43]. Evidence suggests that free biotin is essential for the growth and survival of intestinal microbiota, and its deficiency can disrupt microbial composition, leading to dysbiosis [44]. A Hasan Kadhim et al. [45] found that supplementing the diet of broilers (Ross-308) exposed to oxidative stress with biotin significantly increased average live weight and cumulative feed intake, indicating that biotin can act as an antioxidant, inhibit free radical formation, and improve growth performance—findings consistent with our observation that AMPs promote squab growth. However, this result may be indirect and requires further experimental verification. Beta-tocotrienol, a member of the vitamin E family, exhibits multiple biological activities, including antioxidant and anti-inflammatory properties [46]. By increasing beta-tocotrienol levels, AMPs may help mitigate oxidative stress-induced damage in squabs, maintain cellular and tissue integrity, and thus support healthy growth. Biotin was positively correlated with body weight and ileal VH/CD. As a carboxylase cofactor, it enhances acetyl-CoA carboxylase activity and accelerates fatty acid synthesis [47]. The antioxidant function of beta-tocotrienol protects intestinal epithelial cells from oxidative injury. Together, these two metabolites promote squab growth through a dual mechanism of “energy synthesis and oxidative protection.”

Tryptophan is an essential amino acid involved not only in protein synthesis but also in the biosynthesis of bioactive substances such as serotonin and melatonin [48]. 7-Chloro-L-tryptophan, a tryptophan derivative, reflects the impact of AMPs on amino acid metabolism and neurotransmitter synthesis in squabs. AMPs may alter tryptophan absorption, transport, or metabolic pathways, leading to increased levels of 7-Chloro-L-tryptophan. Given the regulatory roles of serotonin and melatonin in animal growth, behavior, and immunity, this change may exert indirect effects on squab growth performance and physiological status. Future studies should further investigate the underlying mechanisms.

Dihydrozeatin belongs to the cytokinin family and is a plant hormone widely involved in regulating cell division, differentiation, and senescence [49]. Although research on cytokinin-like compounds in animals is limited, emerging evidence suggests that they may influence animal cell proliferation and metabolism by modulating cell cycle proteins and apoptosis-related genes [50]. In this study, dihydrozeatin levels were significantly elevated in the AP group and showed positive correlations with ileal VH/CD and body weight. This suggests that AMPs may enhance dihydrozeatin absorption in the pigeon intestine or activate its endogenous synthesis pathways, resulting in increased metabolite levels. However, this effect may be indirect, and it cannot be ruled out that it is achieved through intermediate pathways such as improving the structure of intestinal villi and enhancing the absorption efficiency of nutrients. Its direct mechanism of action may require further experimental verification.

## 5. Conclusions

In conclusion, supplementing the diet with 200 mg/kg of antimicrobial peptides can significantly enhance the growth performance of squabs, improve liver antioxidant capacity and intestinal tissue morphology. The increase in the abundance of *Lactobacillus* may modulate the intestinal microbiota, potentially benefiting the host through alterations in microbial composition and metabolic profiles. However, it is worth noting that the increase in jejunal MDA levels suggests that while AMPs exert positive effects, they may also have the potential side effect of inducing local oxidative stress.

## Figures and Tables

**Table 1:** Ingredient and nutrient levels of raw grain and pellets in the basal diet (air-dried basis).

Ingredients (%)	Contents
Raw grain	
Corn	25.00
Wheat	5.00
Pea	25.00
Sorghum	5.00
Pellets	
Corn	19.80
Wheat middling	0.80
NaHCO_3_	0.40
Salt	0.40
Yeast powder	2.00
Soybean meal	12.60
CaHPO_4_	0.60
Met	0.16
Lys	0.24
Limestone	0.80
Soybean oil	0.40
Premix ^a^	1.80
Total	100.00
Nutrient components ^b^	
ME/(MJ/kg)	11.65
CP	15.72
Ca	1.20
TP	0.42
Lys	0.77
Met	0.36
Try	0.14

^a^ Premixe provided the following for per kg diet: Biotin 1.2 mg; Folic acid 19 mg; Niacinamide 690 mg; Pantothenic acid 320 mg; VA 200,000–330,000 IU; VD 80,000–165,000 IU; VE 380 IU; VB1 65 mg; VB2,180 mg; VB6 90 mg; Cu 30–830 mg; Fe 2600–25,000 mg; Zn 1500–4000 mg; Mn 1700–5000 mg; I 30–160 mg. ^b^ Nutrient levels were all calculated values.

**Table 2:** Composition of the grit.

Ingredients (%)	Contents
Medium coarse sand	40.00
Yellow mud	18.00
Shell powder	27.50
Calcined gypsum	7.00
Charcoal powder	3.50
Alum	1.50
Gentian herb	1.00
Licorice	1.50
Total	100.00

**Table 3:** Statistical results of metagenomic sequencing.

Group	Sample ID	Raw_Base (G)	Clean_Base (G)	Clean_Q20 (%)	Clean_Q30 (%)	Clean_GC (%)	Effective (%)
AP	G1	7.93	7.79	98.74	96.24	46.28	98.22
	G2	6.71	6.33	97.99	94.53	45.92	94.22
	G3	6.74	6.48	98.21	95.03	44.2	96.04
	G4	6.21	6.07	98.55	95.75	51.92	97.78
	G5	8.06	7.73	98.62	96.32	42.64	95.99
	M1	6.79	6.48	97.95	94.35	43.07	95.45
	M2	6.72	6.29	98.08	94.73	44.39	93.63
	M3	5.98	5.54	97.44	93.21	46.04	92.69
	M4	6.62	6.06	97.38	93.04	43.84	91.59
	M5	6.06	5.68	97.81	94.17	47.06	93.75
CK	G1	6.29	5.76	97.34	93.01	43.77	91.57
	G2	6.02	5.46	97.47	93.19	46.49	90.57
	G3	6.39	6.09	98.22	95.07	43.65	95.33
	G4	6.53	6.44	98.65	95.94	48.53	98.65
	G5	6.98	6.31	97.08	92.25	40.82	90.42
	M1	6.01	5.70	97.93	94.29	44.98	94.78
	M2	16.60	16.07	98.06	94.65	42.79	96.85
	M3	6.92	6.14	96.64	91.28	38.71	88.72
	M4	7.70	6.74	97.36	92.92	45.01	87.61
	M5	7.71	7.60	98.63	95.89	51.36	98.48

Raw_Base: The total number of bases in the raw sequencing data; Clean_Base: The quantity of high-quality bases remaining after filtering; Clean_Q20: The proportion of bases in Clean_Base with a Phred quality score exceeding 20 relative to the total number of bases; Clean_Q30: The proportion of bases in Clean_Base with a Phred quality score exceeding 30 relative to the total number of bases; Clean_GC: The percentage of guanine (G) and cytosine (C) bases among the total base count; Effective: The ratio of high-quality data to raw sequencing data, expressed as a percentage.

**Table 4:** Assembly results of each sample.

Group	Sample ID	Total Len (bp)	Scaftigs Num	Average Len (bp)	N50 Len (bp)	N90 Len (bp)	Max Len (bp)
AP	G1	60,897,103	39,928	1525.17	2705	578	127,556
	G2	623,555,062	630,296	989.31	1031	577	27,385
	G3	814,602,016	739,192	1102.02	1214	615	19,284
	G4	58,100,347	50,407	1152.62	1219	551	117,503
	G5	664,421,876	717,502	926.02	943	569	118,736
	M1	755,528,039	717,186	1053.46	1148	600	93,206
	M2	758,593,231	707,147	1072.75	1172	603	19,734
	M3	311,638,073	320,710	971.71	989	560	30,653
	M4	209,248,151	230,360	908.35	888	545	24,251
	M5	438,374,847	472,598	927.59	927	557	122,336
CK	G1	169,427,565	183,488	923.37	904	544	27,945
	G2	247,413,191	268,260	922.29	912	551	26,724
	G3	839,832,737	763,720	1099.66	1214	620	94,243
	G4	80,963,162	35,355	2290.01	4969	761	299,967
	G5	79,022,972	96,992	814.74	764	529	25,973
	M1	635,843,617	641,034	991.90	1044	579	28,635
	M2	1,125,225,214	231,721	4855.95	11,908	1966	156,136
	M3	78,234,598	101,301	772.30	717	526	16,202
	M4	180,885,066	207,519	871.66	837	539	28,628
	M5	68,863,208	38,378	1794.34	3045	661	279,436

Total len: The total length of all assembled scaftigs; Scaftigs num: The total number of assembled scaftigs; Average len: The mean length of the scaftigs; N50 len: The shortest scaftig length such that the cumulative length of all scaftigs ≥50% of the total assembly length when sorted in descending order; N90 len: The shortest scaftig length such that the cumulative length of all scaftigs ≥90% of the total assembly length when sorted in descending order; Max len: The maximum length among all assembled scaftigs.

## Data Availability

The data supporting the findings of this study are available from the corresponding author upon reasonable request.
